# Absorption Kinetics of Berberine and Dihydroberberine and Their Impact on Glycemia: A Randomized, Controlled, Crossover Pilot Trial

**DOI:** 10.3390/nu14010124

**Published:** 2021-12-28

**Authors:** Jessica M. Moon, Kayla M. Ratliff, Anthony M. Hagele, Richard A. Stecker, Petey W. Mumford, Chad M. Kerksick

**Affiliations:** Exercise and Performance Nutrition Laboratory, School of Health Sciences, College of Science, Technology, and Health, Lindenwood University, St. Charles, MO 63301, USA; jmoon@knights.ucf.edu (J.M.M.); kaylaratliff31@gmail.com (K.M.R.); ahagele@lindenwood.edu (A.M.H.); rstecker@lindenwood.edu (R.A.S.); pmumford@lindenwood.edu (P.W.M.)

**Keywords:** absorption, safety, plants, glucose, insulin

## Abstract

Berberine is a natural alkaloid used to improve glycemia but displays poor bioavailability and increased rates of gastrointestinal distress at higher doses. Recently, dihydroberberine has been developed to combat these challenges. This study was designed to determine the rate and extent to which berberine appeared in human plasma after oral ingestion of a 500 mg dose of berberine (B500) or 100 mg and 200 mg doses of dihydroberberine (D100 and D200). In a randomized, double-blind, crossover fashion, five males (26 ± 2.6 years; 184.2 ± 11.6 cm; 91.8 ± 10.1 kg; 17.1 ± 3.5% fat) completed a four-dose supplementation protocol of placebo (PLA), B500, D100, and D200. The day prior to their scheduled visit, participants ingested three separate doses with breakfast, lunch, and dinner. Participants fasted overnight (8–10 h) and consumed their fourth dose with a standardized test meal (30 g glucose solution, 3 slices white bread) after arrival. Venous blood samples were collected 0, 20, 40, 60, 90, and 120 minutes (min) after ingestion and analyzed for BBR, glucose, and insulin. Peak concentration (C_Max_) and area under the curve (AUC) were calculated for all variables. Baseline berberine levels were different between groups (*p* = 0.006), with pairwise comparisons indicating that baseline levels of PLA and B500 were different than D100. Berberine C_Max_ tended to be different (*p* = 0.06) between all conditions. Specifically, the observed C_Max_ for D100 (3.76 ± 1.4 ng/mL) was different than PLA (0.22 ± 0.18 ng/mL, *p* = 0.005) and B500 (0.4 ± 0.17 ng/mL, *p* = 0.005). C_Max_ for D200 (12.0 ± 10.1 ng/mL) tended (*p* = 0.06) to be different than B500. No difference in C_Max_ was found between D100 and D200 (*p* = 0.11). Significant differences in berberine AUC were found between D100 (284.4 ± 115.9 ng/mL × 120 min) and PLA (20.2 ± 16.2 ng/mL × 120 min, *p* = 0.007) and between D100 and B500 (42.3 ± 17.6 ng/mL × 120 min, *p* = 0.04). Significant differences in D100 BBR AUC (284.4 ± 115.9 ng/mL×120 min) were found between PLA (20.2 ± 16.2 ng/mL × 120 min, *p* = 0.042) and B500 (42.3 ± 17.6 ng/mL × 120 min, *p* = 0.045). Berberine AUC values between D100 and D200 tended (*p* = 0.073) to be different. No significant differences in the levels of glucose (*p* = 0.97) and insulin (*p* = 0.24) were observed across the study protocol. These results provide preliminary evidence that four doses of a 100 mg dose of dihydroberberine and 200 mg dose of dihydroberberine produce significantly greater concentrations of plasma berberine across of two-hour measurement window when compared to a 500 mg dose of berberine or a placebo. The lack of observed changes in glucose and insulin were likely due to the short duration of supplementation and insulin responsive nature of study participants. Follow-up efficacy studies on glucose and insulin changes should be completed to assess the impact of berberine and dihydroberberine supplementation in overweight, glucose intolerant populations.

## 1. Introduction

Berberine is an isoquinoline derivative alkaloid present in various parts (root, stem, fruit, bark) of multiple plants including, in particular, species found in the Coptis, Hydrastis, and Berberis genus [1]. Various animal and culture models have reported on the ability of berberine to exert lipid and glucose-lowering effects in addition to helping with weight loss and improving glucose tolerance [2,3]. For this reason, interest in berberine has suggested it may be beneficial in treating diabetes and obesity. While still being fully characterized, mechanistic actions of berberine consist of it reducing blood lipid levels via action on hepatocyte nuclear factor 1 alpha (HNF-1 alpha) and hepatic LDL receptors, intestinal absorption of cholesterol [4], and glucose levels while increasing insulin sensitivity.

Moreover, berberine is a known activator of AMP Kinase [1,5], which contributes to increases in fatty acid oxidation and a reduction of lipogenic gene expression.

Yin and colleagues [6] published results from two separate studies that both examined the impact of berberine at improving various biomarkers associated with glucose homeostasis. Throughout the first study, berberine (500 mg/dose, 3 doses/day) or metformin was administered over a 3-month period to 36 adults who were recently diagnosed with type 2 diabetes. Levels of hemoglobin A1C, fasting blood glucose, postprandial blood glucose, and triglycerides were all significantly improved in comparison to pre-treatment, and, importantly, the magnitude of changes was similar to what was observed with metformin. An additional investigation was reported where berberine was added to the established treatment plan 48 type 2 diabetes patients. Over a 13-week period, blood glucose (both fasting and postprandial), hemoglobin A1C, fasting, insulin, and HOMA-IR were all improved when berberine was added. These results align with the Perez-Rubio et al. [7] investigation that reported improvements in waist circumference, blood pressure, triglycerides, and area under the curve values for both glucose and insulin (after a 75-g glucose load over a two-hour period) in 24 patients with metabolic syndrome. In this study, berberine was randomly assigned in a double-blind, placebo-controlled fashion at a daily dosage of 1500 mg/day (500 mg/dose, 3 doses/day with meals) for three months. Similar outcomes using berberine have been reported by Rao et al. [8] after completing a randomized, open-label study on 41 diabetic patients where various indicators of glucose and insulin metabolism were improved over the three-month study period.

One challenge associated with oral ingestion of berberine has been its low bioavailability (<1%) reported in both animal [9,10] and human models largely due to poor intestinal absorption and high levels of first-pass removal in the intestines and liver [5]. To overcome this limitation, higher doses of berberine are commonly administered, which has led to a consistent reporting of gastrointestinal adverse events. For example, Yin et al. [6] reported that gastrointestinal adverse events were 34.5% during a 13-week study which provided berberine hydrochloride as a monotherapy or in combination with other treatments. Interestingly all adverse events were reported when berberine was combined with other treatments (metformin, insulin, etc.) and all incidents occurred during the first four weeks of treatment.

For this reason, alternative approaches have been explored to help improve its poor bioavailability. For example, techniques such as encapsulation [11,12], packaging with other nutrients and compounds [13], and spray drying [14] have all been reported on in the literature. In addition, the reduced derivative of berberine, dihydroberberine, has been suggested as a natural alternative to improve bioavailability [15]. Buchanan and investigators [16] examined the ability of transdermal application of dihydroberberine in 40 Sprague-Dawley rats to improve bioavailability, improve glucose homeostasis parameters, and reduce adverse events in comparison to oral berberine gavage and transdermal applications of berberine. Results from both acute and chronic (14 days) pharmacokinetic approaches indicated that transdermal dihydroberberine achieved higher bioavailability than both transdermal and oral berberine administration. Oral administration of dihydroberberine has yet to be reported in human literature, thus initial studies should provide some level of insight into its impact on absorption kinetics, preliminary safety indicators, and adverse events associated with its ingestion. Consequently, the purpose of this study was to conduct a randomized, double-blind, placebo-controlled investigation to examine the dose-dependent impact on absorption kinetics, clinical safety parameters, and adverse events of oral dihydroberberine ingestion in humans.

## 2. Methods

### 2.1. Overview of Research Design

**Figure 1:**
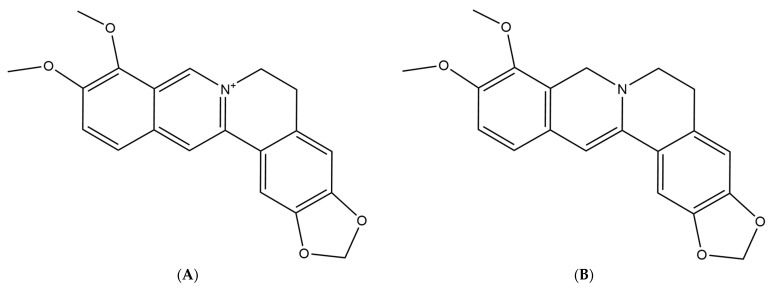
(**A**) Chemical structure of berberine. (**B**) Chemical structure of dihydroberberine.

**Figure 2:**
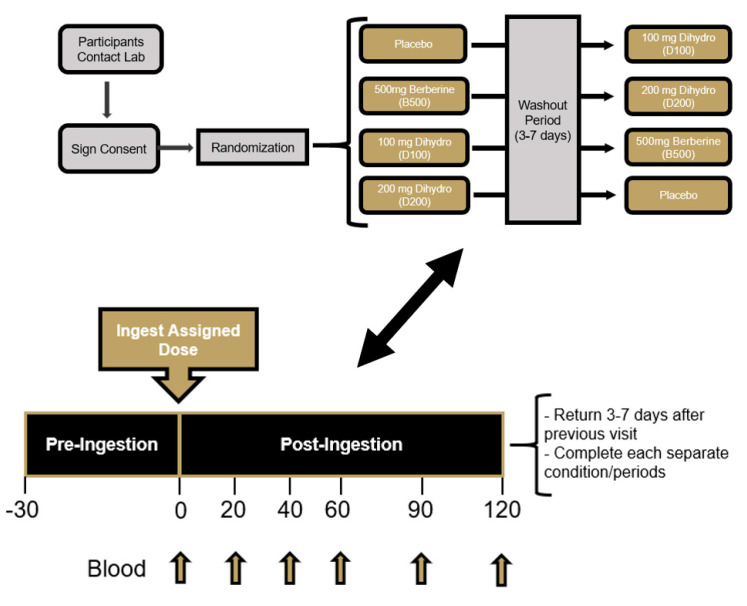
Overview of research design.

This study was completed using a randomized, double-blind, crossover study design. Prior to beginning, all participants signed an IRB-approved informed consent document (Lindenwood University: IRB-20-173, approval date: 22 June 2020) and completed a healthy history questionnaire to determine study eligibility. A power analysis was not computed as this study was considered a proof of concept, pilot investigation. This study protocol and design was retrospectively registered on Clinicaltrials.gov on 25 August 2021 as NCT05021341 (https://clinicaltrials.gov/ct2/show/NCT05021341 accessed on 24 December 2021). All four supplementation conditions were completed by each participant in a crossover fashion with a minimum washout of 72 h. For each supplementation condition, four total doses were consumed with three doses being consumed the day before testing (with breakfast, lunch, and dinner) and the fourth dose being consumed the morning of testing with a standardized test meal after observing an overnight (8 to 10 h) fast (Figure 1). Participants were scheduled to begin each visit at approximately the same times and reported to the laboratory between 600 and 1000 h. Using a randomized, double-blind, placebo-controlled fashion, study participants consumed either a placebo (resistance dextrin) (PLA), 500 mg of berberine (B500), 100 mg dihydroberberine (D100), or 200 mg dihydroberberine (D200). To minimize any order effects from testing, participants were randomized using an online randomization software program (www.random.org accessed on 1 July 2020). Upon arrival for each study visit, participants had their height, body mass, body composition, and hemodynamics (resting heart rate and blood pressure) measured along with an assessment of capillary glucose levels approximately 30 min prior to supplementation ingestion to evaluate fasting status. An indwelling catheter was then implanted into a forearm vein and flushed with saline to remain patent. The fourth and final dose of their assigned supplement was then consumed with 240 milliliters of cold tap water. After supplement ingestion, participants consumed a standardized test meal consisting of a 30-g glucose solution and four slices of white bread. Immediately after ingestion of the test meal, venous blood samples were collected 20, 40, 60, 90, and 120 min (Figure 2). Participants were provided 200 mL of cold water to ingest after each blood collection. Upon processing, all blood samples were stored at −80 °C. Throughout each visit, study participants were asked to report the occurrence of any adverse events (dizziness, headache, nausea, upset stomach, cramping, diarrhea, etc.) that occur throughout completion of the study protocol. All subsequent study visits were completed in an identical fashion and scheduled with at least 72 h between visits.

### 2.2. Study Participants

Prior to participation, all recruited individuals provided signed informed consent using an IRB-approved consent form (Lindenwood University: IRB-20-173, approval date: 22 June 2020). Five healthy males (26.0 ± 2.6 years, 184.2 ± 11.6 cm, 91.8 ± 10.1 kg, 17.1 ± 3.5% fat) successfully completed all study visits (see Table 1 for full participant characteristics). Inclusion criteria included age (18–45 years), healthy and free of disease (as reported by the health screening questionnaire), and physically active (reported at least 30 min of moderate exercise three days a week). Any individual diagnosed with or being treated for cardiac, respiratory, circulatory, musculoskeletal, metabolic, obesity (defined as body mass index > 30 kg/m^2^ and body fat greater than 30%), immune, autoimmune, psychiatric, hematological, neurological, or endocrinological disorder or disease were not allowed to participate in the current study. Any participant taking any dietary supplement besides a multi-vitamin/mineral or a protein supplement were required to abstain from consuming any further doses for 30 days prior to beginning the study protocol.

### 2.3. Supplementation Protocol

Using a randomized, double-blind, placebo-controlled fashion, study participants consumed either a placebo (resistance dextrin) (PLA), 500 mg of berberine (B500), 100 mg dihydroberberine (D100), or 200 mg dihydroberberine (D200). The dihydroberberine was manufactured by NNB Nutrition (Nanjing, China) and is marketed as Glucovantage^®^. Berberine (≥99%) was extracted from *Berberis Aristata* in the hydrochloride form and was used as raw material. Dihydroberberine was obtained by a patented preparation method (CN108997332A). Neither molecule contains any crystalline water. Study participants supplemented consumed four total doses prior to having the absorption kinetics determined. This regimen started the morning before their scheduled visit with participants consuming one dose each with breakfast, lunch, and dinner. In addition to consuming with a meal, all doses were consumed with at least eight fluid ounces of cold tap water. Participants were then required to follow an overnight fast (approximately 8 to 10 h). Upon arrival, capillary glucose levels were determined from a finger-stick blood sample and a handheld glucose monitor (CVS Health Advanced Glucose Monitor, Agamatrix, Inc., Salem, NH, USA). After fasting status was confirmed, the fourth and final dose of their assigned supplement was consumed in front of a study team member with approximately eight fluid ounces of cold tap water. A catheter was then inserted into a forearm vein and approximately 30 min after consuming their final dose a standardized test meal was ingested which consisted of four slices of white bread (approximately 264 kcals, 3.2 g of fat, 50.6 g of carbohydrates and 7.6 g of protein) and 30 g of glucose powder that was mixed with approximately 8–12 fluid ounces of water. The test meal was ingested within five minutes. The ingestion of the test meal was considered to be the 0-min time point and all subsequent measurements occurred as outlined.

All participants were required to complete a supplementation log to document when each dose was consumed. No doses were reported being missed by any participant throughout the study protocol. All study materials were prepared into gelatin capsules of identical size, color, shape, and transparency. All doses were prepared into a single capsule which required each participant to consume a single capsule for each required dose. A resistant dextrin was used as the placebo. All doses were placed into individually labeled bags for each condition for each participant throughout the entire study protocol.

### 2.4. Procedures

#### 2.4.1. Baseline Demographics and Hemodynamics

During the initial visit, after providing written consent, participants were instructed to rest quietly for approximately 10 min before measuring resting heart rate and blood pressure. Resting heart rate was measured by palpating the radial artery for a period of 60 s. While still resting, blood pressure (Omron BP785, Omron Corporation, Kyoto, Japan) measurements were taken before participants resumed normal activity. Participants then had their body mass determined (Tanita BWB-627A, Tokyo, Japan) and recorded to the nearest ±0.1 kg upon arrival prior to each study visit. All recorded body masses were compared to ensure the participant was weight stable. Any participant whose body mass changed by more than 2% between consecutive study visits was excluded from participation. Following body mass measurements, height was measured using a standard wall-mounted stadiometer (Tanita, HR-200, Tokyo, Japan) and recorded to the nearest ±0.5 cm (cm). Fat and fat-free mass was determined using a bioelectrical impedance analyzer (InBody 570, Beverly Hills, CA, USA). Participants were required to observe an overnight fast to ensure an accurate determination of body composition. Body composition analysis occurred between 600 and 1000 h by trained research personnel. All assessments were completed according to device specifications.

#### 2.4.2. Dietary Monitoring

Prior to their baseline visit, study participants completed a hand-written four-day food record (three weekdays and one weekend day). The four-day food log was provided to allow participants to replicate their diets. In addition to the four-day food log, participants were instructed on how to complete the ASA24 online dietary assessment tool (https://epi.grants.cancer.gov/asa24/ accessed on 23 December 2021), for determination of baseline caloric and macronutrient intake. From this information, study participants were asked to replicate their dietary intake prior to each subsequent testing visit. Study participants reported 100% compliance to this protocol.

#### 2.4.3. Venous Blood Collection and Processing

To confirm a fasting state prior to catheter insertion and supplement administration, capillary blood was collected from a finger on their non-dominant hand. The collected blood was analyzed using a hand-held glucose analyzer (CVS Health Advanced Glucose Monitor, Agamatrix, Inc., Salem, NH, USA). Fasting was confirmed if glucose was <110 mg/dL. Within each supplementation condition, study participants had their venous blood collected on six different occasions: 0, 20, 40, 60, 90, and 120 min after ingestion of their assigned study agent. Blood was collected via a forearm vein using standard phlebotomy techniques into serum and ethylenediaminetetraacetic acid EDTA-coated Vacutainer™ tubes. To establish safety, whole blood samples were collected into EDTA and serum tubes for assessment of complete blood count and comprehensive metabolic count, respectively. Blood samples collected into EDTA were gently inverted ten times while blood samples collected into serum tubes were gently inverted ten times and allowed to clot at room temperature. For same day analysis, these samples were placed into a chilled container and sent to a commercial diagnostic laboratory (Quest Diagnostics). Plasma samples used for berberine determination were collected into EDTA tubes, gently inverted ten times and centrifuged at 3000 revolutions per minute (rpm) (MegaFuge XFR, Thermo Fisher Scientific, Waltham, MA, USA) at 4 °C for 20 min. After centrifugation, 400 μL aliquots of plasma were removed and frozen at −80 °C within four hours of collection.

#### 2.4.4. Complete Blood Count

To assess adverse events reported in response to the assigned supplementation, whole blood samples were collected in EDTA tubes and analyzed at a commercial diagnostic laboratory (Quest Diagnostics) for changes in red blood cell count, white blood cell count, platelet count, hemoglobin, hematocrit, red blood cell dimension width (RDW), mean corpuscle volume (MCV), mean corpuscle hemoglobin (MCH), mean corpuscle hemoglobin content (MCHC), neutrophil % and cell count, lymphocytes % and cell count, monocytes % and cell count, eosinophils % and cell count, and basophils % and cell count (granulocytes → neutrophils, eosinophils, basophils). Complete blood counts were only assessed at the 0 and 120-min time points.

#### 2.4.5. Comprehensive Metabolic Panel

To assess adverse events in response to the assigned supplementation, serum samples were assayed at a commercial diagnostic laboratory (Quest Diagnostics) for a comprehensive metabolic panel (albumin, albumin/globulin ratio (calculated), alkaline phosphatase, ALT, AST, BUN/creatinine ratio (calculated), calcium, carbon dioxide, chloride, creatinine with estimated GFR, globulin, glucose, potassium, sodium, total bilirubin, total protein, and urea nitrogen). Comprehensive metabolic panels were only assessed at the 0 and 120-min time points.

#### 2.4.6. Glucose and Insulin Determination

Each plasma sample was analyzed in duplicate for glucose and insulin concentration. Plasma glucose was analyzed at a commercial diagnostic laboratory (Quest Diagnostics) using automated clinical analyzers while insulin concentrations were assessed using a standard ELISA technique (DRG International, EIA2935, Springfield, NJ, USA). Briefly, all standards, samples, and controls were analyzed in duplicate across a range of 1.76–100 μIU/mL. Plate to plate controls were employed between all analyzed plates and exhibited a 6.3% coefficient of variation between the plates.

#### 2.4.7. *Plasma Berberine Determination*

Berberine analysis was performed in plasma samples by Heartland Assays (Iowa State University Research Park, Ames, IA, USA) using coupled liquid chromatography-mass spectrometry-mass spectrometry technology (LC-MS-MS) following the procedures of Buchanan et al. [16]. Briefly, BBR and d_6_-BBR (Sigma-Aldrich, St. Louis, MO, USA and Toronto Research Chemicals, Toronto, Canada, respectively) standards were prepared using gravimetric determination and were dissolved in 50:50 methanol: water. As previously published, plasma samples and controls were removed from freezer storage and allowed to thaw at room temperature. An amount of 100 µL of plasma was transferred to a 13 × 100 glass test tube followed by the addition of 15.0 µL of d_6_-BBR internal standard (ISTD, 0.5 ng/µL). Next, samples were treated with 400 µL of acetonitrile containing 3% acetic acid, vortexed and centrifuged. The supernatant was transferred to 12 × 75 glass test tube and dried in a vacuum dryer. A standard curve (0.3–1000.0 ng/mL) was prepared and dried. Samples, controls, and standard curve were reconstituted in 50 µL of LC/MS/MS organic and 50 µL aqueous mobile phase solutions (Mobile phase B, LCMS-grade methanol and Mobile phase A, LCMS-grade water, both containing 0.2% formic acid and 0.1% 10 M ammonium formate solution) vortexed and transferred into LC/MS/MS vials. Chromatographic separation of BBR and d_6_-BBR was conducted on an Agilent Poroshell C18 column (2.1 × 50 mm, 2.7 µm) using an Agilent 6460 triple quadrupole LC/MS/MS system (Santa Clara, CA, USA). The initial gradient conditions were 45% B from 0 to 2 min and advanced to 95% B over 1 min with a flow rate of 0.40 mL/minute. BBR and d_6_-BBR data were collected using the Multiple Reaction Monitoring (MRM) mode with Mass Hunter acquisition software (Agilent, Santa Clara, CA, USA). The MRM pairs used for BBR were 337.1 → 322.1 and 337.1 → 293.1 and 343.1 → 325.2 and 343.1 → 307.1 for d_6_-BBR. Mass Hunter Quantitation software was used to quantitate the unknown plasma samples based on best fit standard curves. Quality control samples were run at two levels in each assay (4.0 and 100.0 ng/mL). Average recovery was 102% and ranged from 95% to 108%. The average accuracy (bias from nominal) was 1.0%. The average precision (CV) was 2.1% and 1.5% for intra- and inter-assay precision, respectively.

### 2.5. Statistical Analysis

The primary outcome for this study protocol was berberine AUC while secondary outcomes were considered to the maximum berberine concentration (C_Max_) as well as the AUC and C_Max_ for glucose and insulin. Area under the curve (AUC) for berberine, glucose, and insulin were calculated using the trapezoidal method (Equation (1)), where *C*_0_ is the unknown concentration at the first time point of interest, *C*_1_ is the unknown concentration at the second time point of interest, and *Time*_1–0_ is the total time interval between the two time points when the unknown was measured. These individual AUC values for each time interval were then summed to determine the total AUC for berberine, glucose, and insulin (Equation (2)).

(1) $$AUC_{1}=\frac{C_{0}+C_{1}}{2}\times Time_{1-0}$$

(2) $$AUC_{Total}=AUC_{1}+AUC_{2}+AUC_{3}+\ldots +AUC_{n}$$

All analyses were completed using Microsoft Excel and the Statistical Package for the Social Sciences (v26; SPSS Inc., Chicago, IL, USA) while provided figures were developed using GraphPad (La Jolla, CA, USA). For all dependent measures, descriptive statistics are presented herein as mean ± standard deviations. Before any statistical tests were completed, normality was assessed using a Shapiro-Wilk test for all dependent variables. All non-normal data were transformed using natural logarithms, cubed, and then square root transformation approaches and was retested for normality. Data were then analyzed using both parametric and non-parametric approaches. In all such situations, the final statistical decision was identical whether parametric or non-parametric approaches were completed. All reported *p*-values are computed using parametric approaches. Blood markers were analyzed with a mixed factorial ANOVA, and if a significant interaction was observed then follow-up simple effects testing was performed using a repeated measures ANOVA followed by post-hoc testing consisting of paired samples *t*-tests. Body mass, blood pressure, AUC, and C_Max_ variables were analyzed with a repeated measures ANOVA followed by post-hoc testing consisting of paired samples t-tests. For all statistical tests, data were considered statistically significant when the probability of type I error was 0.05 or less. Trends were highlighted and discussed when *p*-values were between 0.05 and 0.10.

## 3. Results

### 3.1. Pre-Supplementation Status

Table 2 outlines the pre-supplementation values for body mass and hemodynamics. Body mass was significantly different between groups (*p* = 0.045), and post-hoc testing revealed differences between PLA (90.6 ± 10.1 kg) and D200 (91.4 ± 9.7 kg). Resting heart rate (*p* = 0.21), systolic blood pressure (*p* = 0.07), and diastolic blood pressure (*p* = 0.87) were not different.

### 3.2. Protocol Compliance

Compliance to the supplementation regimen was 100% with all participants reporting that all supplements were ingested as instructed with the final dose being ingested in front of investigators. All study participants were asked to record their food and fluid intake for two days. Compliance was reported at 87.5% as one participant did not record their food and fluid intake on one of the days. Dietary intake is provided in Table 1.

### 3.3. Adverse Events

A total of 11 adverse events were reported throughout the study protocol by two or 40% of the study participants. The largest number of adverse events (*n* = 6) were reported in D100, *n* = 3 were reported in PLA, *n* = 1 for D200, and *n* = 1 for B500. All but one reported adverse event (10/11 or 90.9%) was deemed by study participants to be mild in severity and one being moderate severity (See Table 3).

### 3.4. Berberine

Berberine data were not-normally distributed. The data were transformed using the aforementioned methods and reassessed for normality, which was unable to be achieved due to two high values in the D200 condition at the 60-min timepoint. Subsequently, all berberine data were analyzed using parametric approaches. A significant group x time interaction was revealed for berberine (*p* = 0.05). Simple main effects tests revealed no changes over time within each condition (*p* > 0.05), but significant changes between conditions at the following time points: baseline (*p* = 0.006), 20-min (*p* < 0.001), and 40-min (*p* = 0.039). At baseline, both D100 (2.60 ± 1.06 ng/mL) and D200 (4.54 ± 2.24 ng/mL) conditions had higher berberine values compared to placebo (0.14 ± 0.09 ng/mL; D100, *p* = 0.007; D200, *p* = 0.011) and B500 (0.30 ± 0.12 ng/mL; D100, *p* = 0.006; D200, *p* = 0.013). At the 20-min time point, both D100 (3.00 ± 1.85 ng/mL) and D200 (6.06 ± 2.50 ng/mL) conditions had higher berberine values compared to placebo (0.18 ± 0.18 ng/mL; D100, *p* = 0.030; D200, *p* = 0.005) and B500 (0.32 ± 0.20 ng/mL; D100, *p* = 0.008; D200, *p* = 0.029), but D200 had higher berberine values compared to D100 (*p* = 0.050). Lastly, at 40 min both D100 (2.32 ± 1.27 ng/mL) and D200 (6.90 ± 4.94 ng/mL) conditions had higher berberine values compared to placebo (0.20 ± 0.14 ng/mL; D100, *p* = 0.018; D200, *p* = 0.037) and B500 (0.36 ± 0.15 ng/mL; D100, *p* = 0.022; D200, *p* = 0.040), but D200 had higher berberine values compared to D100 (*p* = 0.050). There was a significant main effect for condition (*p* = 0.04), but no significant main effect for time (*p* = 0.18) was observed.

**Figure 3:**
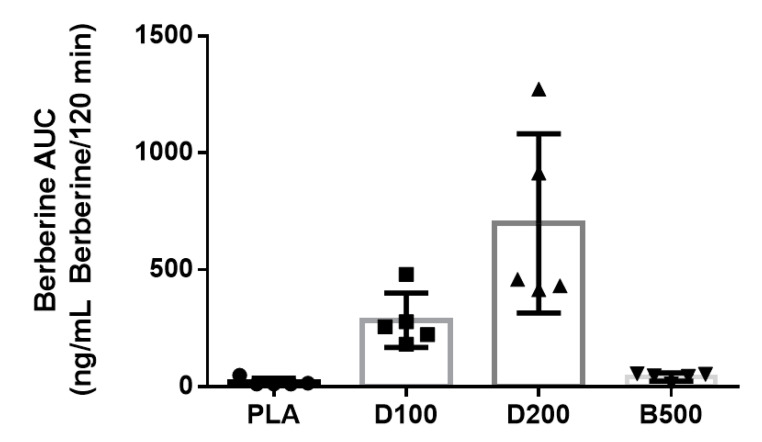
Individual and aggregated mean values for berberine AUC by condition.

Berberine AUC values (Figure 3) were significantly difference between conditions (*p* = 0.045). Follow-up analysis indicated that higher berberine AUC values were identified in D200 (929 ± 694 ng/mL berberine/120 min) when compared to PLA (20.2 ± 16.2 ng/mL Berberine/120 min, *p* = 0.042) and B500 (42.3 ± 17.5 ng/mL Berberine/120 min, *p* = 0.045). Significantly higher berberine AUC values were identified for D100 (284.2 ± 115.9 ng/mL Berberine/120 min) when compared to placebo (20.2 ± 16.2 ng/mL Berberine/120 min, *p* = 0.007) and B500 (42.3 ± 17.5 ng/mL Berberine/120 min, *p* = 0.007). Berberine AUC values between D100 (284.2 ± 115.9) and D200 (929 ± 694 ng/mL Berberine/120 min) approached statistical significance (*p* = 0.07). Berberine AUC values between PLA (20.2 ± 16.2 ng/mL Berberine/120 min) and B500 (42.3 ± 17.5 ng/mL Berberine/120 min) tended (*p* = 0.096) to be different. Berberine C_Max_ values tended to be different between conditions (*p* = 0.064). Further, differences in C_Max_ between D100 and PLA were significant *p* = 0.005) while differences between PLA and D200 (*p* = 0.06) as well as PLA and B500 (*p* = 0.09) tended to be different.

Each individual symbol represents the individual AUC response for one study participant. For all graphs below, *n* = 5 participants completed each condition. PLA = Placebo; D100 = 100 mg dose of dihydroberberine; D200 = 200 mg dose of dihydroberberine; B500 = 500 mg dose of berberine. Data are presented as mean ± SD.

### 3.5. Glucose

There was no significant group x time interaction (*p* = 0.97) for glucose measures. However, a significant time effect (*p* < 0.001) was observed where glucose concentrations were significantly elevated at the 40-min timepoint (129.25 ± 27.43 mg/dL) compared to the baseline timepoint (91.05 ± 5.61 mg/dL, *p* < 0.022). Furthermore, both the 20-min timepoint (119.5 ± 21.27 mg/dL) and the 40-min timepoint (129.25 ± 27.43 mg/dL) had elevated glucose concentrations compared to the 90-min timepoint (85.40 ± 18.15 mg/dL; 20 min, *p* = 0.045; 40 min, *p* = 0.007), and the 120-min timepoint (69.6 ± 10.19 mg/dL; 20 min, *p* = 0.044; 40 min, *p* < 0.001). The 60-min timepoint (105.45 ± 23.55 mg/dL) had elevated glucose concentrations compared to the 120-min timepoint (69.60 ± 13.59 mg/dL, *p* = 0.033). Both AUC and C_Max_ values were calculated for all four conditions. Oneway ANOVA indicated no significant differences were observed for AUC (*p* = 0.92) and C_Max_ (*p* = 0.77) values (See Table 4 and Table 5).

### 3.6. Insulin

There was no significant group x time interaction (*p* = 0.095) for insulin measures. A significant time effect (*p* < 0.001) was observed where insulin concentrations were significantly elevated at 20 min (50.8 ± 20.7 µIU/mL, *p* = 0.02), 40 min (93.0 ± 18.2 µIU/mL, *p* = 0.02, and 60 min (82.9 ± 13.6 µIU/mL, *p* = 0.003) in comparison to baseline (16.3 ± 6.1 µIU/mL). Additionally, insulin concentrations were significantly higher at 40 min (93.0 ± 18.2 µIU/mL) and 60 min (82.9 ± 13.6 µIU/mL) compared to 20 min (50.8 ± 20.7 µIU/mL; 40 min, *p* = 0.002; 60 min, *p* = 0.018) and 120 min (45.1 ± 24.5 µIU/mL; 40 min, *p* = 0.002; 60 min, *p* = 0.013). No significant condition effect (*p* > 0.05) was identified. No significant differences were observed for AUC (*p* = 0.22) and C_Max_ (*p* = 0.36) insulin values (See Table 4 and Table 5).

### 3.7. Whole Blood and Serum Markers

Complete blood count analysis was completed on collected whole-blood samples to assess safety and presence of any adverse responses to supplementation. As seen in Table 6, absolute lymphocytes analysis revealed a significant main effect of condition (*p* = 0.011) where D100 had elevated concentrations compared to D200 (*p* = 0.032) but were in clinically accepted norms. Absolute monocytes analysis revealed a significant group × time interaction (*p* = 0.047) and follow-up simple main effects testing revealed significant changes over time within the Placebo (*p* = 0.042) and D100 (*p* = 0.029) conditions. Specifically, at 120 min within the Placebo and D100 conditions absolute monocytes were higher than baseline albeit they were within clinically accepted norms. Furthermore, no significant changes between conditions were revealed at any time point for absolute monocytes (*p* > 0.05). All other variables for complete blood count did not change over time, between conditions, or reveal any interactions (*p* > 0.05).

Comprehensive metabolic panels were analyzed in collected serum samples. As seen in Table 7, analysis revealed significant main effects of time for glucose (*p* = 0.024), sodium (*p* < 0.001), potassium (*p* = 0.007), and chloride (*p* = 0.024) and these changes were within clinically accepted norms. A significant group × time interaction was revealed for creatinine (*p* = 0.008), and follow-up simple main effects testing revealed significant changes over time within the D100 (*p* = 0.031) and D200 (*p* = 0.030) conditions. Within the D100 condition at 120 min, creatinine measures were higher than baseline, and within the D200 condition, baseline measures were higher than 120 min, however, these measures are within clinically accepted norms. Additionally, there were significant creatinine differences between conditions at baseline (*p* = 0.039) and 120 min (*p* = 0.009) measures. At baseline, D200 (*p* = 0.030) and B500 (*p* = 0.016) conditions had higher creatinine measures compared to D100 and these differences were within clinically accepted norms. Lastly, a significant main effect of condition was observed for creatinine measures (*p* = 0.040) where D100 was higher than placebo (*p* = 0.035) but was within clinically accepted norms.

A significant group x time interaction was revealed for globular filtration rate (*p* = 0.016) and follow-up simple main effects testing revealed significant changes over time within the D100 (*p* = 0.047) and D200 (*p* = 0.037) conditions. Within the D100 condition, baseline globular filtration rate measures were higher than 120 min, and within the D200 condition 120 min measures were higher than baseline, however, these measures are within clinically accepted norms. Additionally, follow-up simple main effects testing from the interaction revealed significant differences between conditions for globular filtration rate measures at baseline (*p* = 0.050) and 120 min (*p* = 0.005). At baseline, globular filtration rate measures were higher in D100 compared to D200 (*p* = 0.048) and B500 (*p* = 0.038), which were within clinically accepted norms. At 120 min, globular filtration rate measures were higher in D200 (*p* = 0.024), D100 (*p* = 0.024), and B500 (*p* = 0.047) compared to placebo and were within clinically accepted norms. Lastly, a significant main effect of condition was observed for globular filtration rate measures (*p* = 0.033) where D100 was higher than placebo (*p* = 0.028) but was within clinically accepted norms.

A significant group x time interaction was revealed for calcium (*p* = 0.049) and follow-up simple main effects testing revealed significant changes over time within the placebo condition (*p* = 0.009), the levels of which were higher at 120 min compared to baseline, but within clinically accepted norms. Furthermore, a follow-up simple main effects revealed significant differences between conditions for calcium measures at 120 min (*p* = 0.030) where placebo was higher than D200 (*p* = 0.040) and all measures were within clinically accepted norms. All other variables for the comprehensive metabolic panel did not change over time, between conditions, or reveal any interactions (*p* > 0.05).

## 4. Discussion

The intent of this project was to evaluate the observed differences in plasma berberine concentrations after oral ingestion of two doses (100 mg [D100] and 200 mg [D200], respectively) of dihydroberberine and one dose (500 mg) of berberine (B500). Findings from this pilot approach revealed that area under the curve calculations for berberine were significantly greater when either dose of dihydroberberine was ingested in comparison to the values observed for berberine and placebo (PLA). In addition, peak berberine concentrations (C_Max_) tended to be different between all conditions. Specifically, the 100 mg dose of dihydroberberine was significantly greater than peak values observed in PLA and B500 while peak concentrations for the 200 mg dose of dihydroberberine tended to be different than what was observed in the berberine group.

Of the available human research that has explored efficacy and safety of oral berberine ingestion, the majority of these studies have employed a dosing regimen that consists of two to three daily doses with meals (to reduce incidence of gastrointestinal complications) at a dosage of 500 mg per dose. For example, Perez-Rubio et al. [7] had 24 metabolic syndrome patients randomly ingest, in a double-blind, placebo-controlled fashion, three daily doses of 500 mg/dose (total daily dosage of 1500 mg/day) for three months. Similarly, Yin et al. [6] also randomly assigned type 2 diabetes patients to ingest three daily 500 mg doses for a total of 13 weeks. Finally, Zhang et al. [17] randomly assigned 116 individuals diagnosed with type 2 diabetes to consume two 500 mg daily doses (1000 mg total) over a 3-month period. While results of these studies outline efficacy outcomes in various clinical populations, very limited human data are available that has outlined the absorption pattern and plasma kinetics after oral berberine ingestion. In this respect, it has been previously reported that oral bioavailability of berberine is extremely low (<1%) in both animal [9,10] and human models largely due to poor intestinal absorption and high levels of first-pass removal in the intestines and liver [5]. This has led to the identification of various strategies to increase bioavailability, including administration of dihydroberberine. Currently, no human research has appeared in the peer-reviewed literature that has examined oral ingestion of dihydroberberine. Towards this end, Buchanan et al. [16] compared the pharmacokinetics associated with transdermal administration of berberine and dihydroberberine in Sprague-Dawley rats. Using this model and over an 8-h period, transdermal application of dihydroberberine resulted in greater AUC values than what was observed for both transdermal and oral administration of berberine. Moreover, transdermal delivery of berberine resulted in greater AUC values than what was observed for oral administration, further highlighting the poor bioavailability of oral berberine ingestion.

Other highlights from the present study include the statistically significant higher outcomes at baseline (after three doses, but prior to the 4th dose in the present study) in D100 and D200 when compared to PLA and B500. When viewed in comparison to PLA, this outcome was not surprising and some might deem it expected considering that dihydroberberine was administered, however, the finding of greater levels in comparison to berberine seemingly speaks to the ability of dihydroberberine to maintain berberine levels in the blood better than oral berberine ingestion. Whether or not these increases in plasma berberine translate beyond one day or measurement or improvements in efficacy outcomes commonly associated with berberine supplementation cannot be determined from this project. In this respect, more research is certainly needed with dihydroberberine to determine how well the greater plasma berberine levels improve dyslipidemia and glucose and insulin homeostasis as commonly reported in the literature.

A key limitation with the present study is the sample size on this project. While the fully randomized, crossover, double-blind, and placebo-controlled approach was a sound approach for this pilot assessment, it is readily acknowledged by the authors that more study participants are needed on future studies to continue to establish the safety and efficacy of berberine and dihydroberberine. Further to the point and beyond just having a low sample size, one needs to closely consider our plasma berberine data as two participants in the D200 at the 60-min time point exhibited high values, which nearly achieved traditional definitions of being a statistical outlier (>±3 of standardized residual estimates). When combined with the low sample size, these data exerted substantial impact on the distribution of these data. As such, traditional approaches to transforming the data were unsuccessful at achieving normality and all data were subsequently analyzed using a parametric approach. Furthermore, all analysis was also completed using an intent-to-treat approach with those values removed and the high values replaced with the previous data point and no meaningful differences were noted in the outcomes of the project. Another limitation might be considered to be the small number of doses ingested prior to assessing the absorption kinetics. This was an a priori consideration as our primary focus for this pilot project was to assess and compare the absorption kinetics of two different doses of oral dihydroberberine in comparison to oral ingestion of berberine and placebo.

## 5. Conclusions

In conclusion, results from this study represent what we believe are the first data in humans to evaluate the absorption kinetics of berberine and two doses of dihydroberberine. Results from this study demonstrate that dihydroberberine, irrespective of what dose is delivered, achieves greater area under the curve as well as peak berberine concentrations when compared to oral ingestion of berberine or placebo. Since berberine use has traditionally spanned several months to establish the observed efficacy outcomes known for berberine (improved glucose and insulin homeostasis and lipid parameters), several follow-up approaches are recommended. First, can the increased levels of berberine seen from dihydroberberine ingestion usher in faster improvements in outcomes of interest. In a similar vein, are the outcomes improved as a result of dihydroberberine ingestion in comparison to berberine ingestion.

## Figures and Tables

**Table 1:** Baseline age, gender, height (cm), weight (kg), body mass index, % fat, heart rate, systolic blood pressure, diastolic blood press, energy, carbohydrates, proteins, and fat intake.

	Mean	SD	Minimum	Maximum
Age	26.0	2.6	23	30
Height (cm)	184.2	11.6	171	202
Weight (kg)	91.8	10.1	82.4	108.5
Body mass index (kg/m^2^)	27.1	3.9	22.6	33.1
% fat	17.1	3.5	12.5	20.9
Heart rate (beats/minute)	61.0	12.0	41	70
Systolic blood pressure (mm Hg)	122.3	11.4	111	138
Diastolic blood pressure (mm Hg)	72.4	8.8	64	70
Energy intake (kcals/day)	2433	810	1503	3695
Carbohydrate intake (grams/day)	217	62	144	312
Protein intake (grams/day)	121	34	66	153
Fat intake (grams/day)	112	45	75	186

**Table 2:** Baseline body mass, hemodynamic, glucose, insulin, and berberine.

Raw Data	Group	Pre	*p*-Value
Body Mass (kg)	PLA	90.6 ± 10.1 ^‡^	0.043
	D100	91.6 ± 10.3	
	D200	91.4 ± 9.7	
	B500	90.8 ± 10.0	
Resting Heart Rate (beats/minute)	PLA	64 ± 10	0.21
	D100	62 ± 6	
	D200	60 ± 5	
	B500	57 ± 9	
Systolic Blood Pressure (mm Hg)	PLA	120 ± 8	0.07
	D100	115 ± 10	
	D200	118 ± 11	
	B500	124 ± 10	
Diastolic Blood Pressure (mm Hg)	PLA	72 ± 5	0.87
	D100	70 ± 5	
	D200	70 ± 10	
	B500	72 ± 7	
Glucose (mg/dL)	PLA	91.8 ± 7.6	0.59
	D100	92.0 ± 8.5	
	D200	90.6 ± 3.4	
	B500	89.8 ± 1.8	
Berberine (ng/mL)	PLA	0.14 ± 0.09 ^†,‡^	0.006
	D100	2.60 ± 1.06	
	D200	4.54 ± 2.24	
	B500	0.30 ± 0.12 ^†,‡^	
Insulin (μIU/mL)	PLA	15.4 ± 5.4	0.20
	D100	16.7 ± 9.1	
	D200	14.8 ± 4.4	
	B500	18.4 ± 6.3	

^†^ = Different than D100 (*p* < 0.05); ^‡^ = Different than D200 (*p* < 0.05).

**Table 3:** Adverse event table.

	PLA	D100	D200	B500
*n*	5	5	5	5
# of AE’s Reported				
Mild	3	5	1	1
Moderate	0	1	0	0
Severe	0	0	0	0
	Adverse Events Breakdown			
Gastrointestinal				
Diarrhea	0	0	0	0
Nausea	0	2	0	0
Upset stomach	0	0	1	0
Stomach cramping	2	0	0	0
Pain				
Headache	0	2	0	1
Cardiovascular				
Abnormal heart rhythm	1	0	0	0
Dizziness	0	2	0	0
Constitutional Symptoms				
Nervousness	0	0	0	0
Blurred vision	0	0	0	0
Summary				
Total Adverse Events	3	6	1	1
# of Subjects Reporting AE	2	2	1	1
% of Subjects Reporting AE	40%	40%	20%	20%

PLA = Placebo; D100 = 100 mg dose of dihydroberberine; D200 = 200 mg dose of dihydroberberine; B500 = 500 mg dose of berberine.

**Table 4:** Berberine (ng/mL), glucose (mg/dL), and insulin (μIU/mL) data.

Berberine (ng/mL)	0 min	20 min	40 min	60 min	90 min	120 min		*p*
PLA	0.14 ± 0.09	0.18 ± 0.18	0.20 ± 0.14	0.16 ± 0.13	0.16 ± 0.13	0.16 ± 0.13	Group	0.002
B500	0.30 ± 0.12	0.32 ± 0.20	0.36 ± 0.15	0.38 ± 0.16	0.39 ± 0.16	0.32 ± 0.13	Time	0.18
D100	2.6 ± 1.1 ^†,‡^	3.0 ± 1.9 ^†,‡^	2.3 ± 1.3 ^†,‡^	2.5 ± 1.0	2.1 ± 0.8	1.9 ± 0.9	G x T	0.050
D200	4.5 ± 2.2 ^†,‡^	6.1 ± 2.5 ^†,‡,^*	6.9 ± 4.9 ^†,‡,^*	11.4 ± 10.7	7.8 ± 6.5	7.2 ± 6.5		
**Glucose (mg/dL)**	**0 min**	**20 min**	**40 min**	**60 min**	**90 min**	**120 min**		***p***
PLA	91.8 ± 7.6	120.4 ± 25.4	129.6 ± 33.0	102.8 ± 27.6	92.0 ± 23.9	63.4 ± 9.3	Group	0.92
B500	89.8 ± 1.8	116.4 ± 30.1	125.6 ± 35.7	101.8 ± 30.9	84.0 ± 25.1	73.0 ± 13.7	Time	<0.001
D100	92.0 ± 8.5	121.8 ± 20.9	126.8 ± 24.5	106.4 ± 14.9	81.2 ± 10.1	69.4 ± 7.9	G x T	0.97
D200	90.6 ± 3.4	119.2 ± 11.8	135.0 ± 23.3	110.8 ± 25.2	84.4 ± 13.4	72.6 ± 21.6		
**Insulin** **(μIU/mL)**	**0 min**	**20 min**	**40 min**	**60 min**	**90 min**	**120 min**		***p***
PLA	15.4 ± 5.4	47.9 ± 31.3	106.9 ± 26.8	85.0 ± 21.0	78.5 ± 23.5	35.4 ± 19.2	Group	0.43
B500	18.4 ± 6.3	38.4 ± 26.9	87.1 ± 24.5	78.1 ± 23.0	58.0 ± 23.5	58.0 ± 23.5	Time	<0.001
D100	16.7 ± 9.1	47.2 ± 17.6	93.0 ± 35.2	84.5 ± 33.2	60.9 ± 23.1	34.6 ± 14.8	G x T	0.10
D200	14.8 ± 4.4	69.6 ± 28.6	85.0 ± 9.0	83.9 ± 27.4	78.9 ± 12.6	53.3 ± 29.6		

^†^ = Different than placebo (*p* < 0.05); ^‡^ = Different than B500 (*p* < 0.05); * = Different than D100 (*p* < 0.05).

**Table 5:** Area under the curve (AUC) and concentration max (C_Max_) for berberine, insulin, and glucose.

Berberine	AUC (ng/mL × 120 min)	C_Max_ (ng/mL)
PLA	20.2 ± 16.2	0.22 ± 0.18
B500	42.3 ± 17.6	0.40 ± 0.17
D100	284.2 ± 115.9	3.8 ± 1.4
D200	929 ± 694	12.0 ± 10.1
*p*	0.045	0.06
**Insulin**	**AUC** **(μIU/mL × 120 min)**	**C_Max_** **(μIU/mL)**
PLA	8260 ± 420	110.6 ± 24.3
B500	7429 ± 1519	99.1 ± 6.6
D100	8502 ± 717	107.2 ± 21.4
D200	7242 ± 1263	98.1 ± 3.4
*p*	0.22	0.36
**Glucose**	**AUC** **(mg/dL × 120 min)**	**C_Max_** **(mg/dL)**
PLA	12,199 ± 1602	146.2 ± 13.9
B500	12,029 ± 194	139.4 ± 32.8
D100	12,381 ± 1128	140.8 ± 8.8
D200	11,898 ± 2058	139.0 ± 16.6
*p*	0.92	0.77

**Table 6:** Complete blood count.

Variable Name	Group	Pre	Post	Group (G) (*p*)	Time (T) *(p)*	G x T (*p*)
White Blood Cell Count (10^3^ × cells/μL)	PLA	5.6 ± 1.0	5.7 ± 1.3	0.74	0.45	0.20
	D100	5.7 ± 1.1	5.9 ± 1.3			
	D200	6.6 ± 2.4	6.2 ± 2.1			
	B500	5.8 ± 1.2	5.3 ± 1.2			
Red Blood Cell Count (10^3^ × cells/μL)	PLA	4.8 ± 0.2	4.8 ± 0.1	0.10	0.54	0.30
	D100	4.7 ±0.3	4.7 ± 0.3			
	D200	4.7 ± 0.3	4.7 ± 0.2			
	B500	5.0 ± 0.3	4.8 ± 0.4			
Hemoglobin (g/dL)	PLA	14.8 ± 0.5	14.8 ± 0.7	0.15	0.47	0.20
	D100	14.3 ± 0.4	14.5 ± 0.5			
	D200	14.3 ± 0.6	14.2 ± 0.4			
	B500	15.2 ± 1.0	14.8 ± 0.8			
Hematocrit (%)	PLA	43.2 ± 0.8	43.4 ± 1.4	0.20	0.73	0.19
	D100	42.2 ± 0.7	42.6 ± 1.4			
	D200	42.1 ± 1.3	42.0 ± 0.4			
	B500	44.6 ± 2.8	43.7 ± 3.0			
Mean Corpuscular Volume	PLA	89.4 ± 3.5	90.2 ± 3.8	0.87	0.44	0.71
	D100	90.1 ±4.7	90.0 ± 4.3			
	D200	90.4 ± 3.2	90.4 ± 3.8			
	B500	90.2 ± 2.8	90.5 ± 3.7			
Mean Corpuscular Hemoglobin	PLA	30.6 ± 1.6	30.7 ± 1.5	0.86	0.88	0.81
	D100	30.6 ± 1.6	30.5 ± 1.6			
	D200	30.7 ± 1.3	30.6 ± 1.6			
	B500	30.6 ± 1.6	30.7 ± 1.4			
Mean Corpuscular Hemoglobin Content	PLA	34.2 ± 0.6	34.1 ± 0.8	0.91	0.43	0.93
	D100	34.0 ± 0.6	34.0 ± 0.5			
	D200	34.0 ± 0.8	33.8 ± 0.7			
	B500	34.0 ± 0.9	33.9 ± 0.7			
Red Cell Distribution Width (%)	PLA	12.1 ± 0.4	12.0 ± 0.5	0.93	0.08	0.55
	D100	12.1 ± 0.4	12.0 ± 0.5			
	D200	12.1 ±0.4	12.1 ± 0.4			
	B500	12.0 ± 0.4	12.0 ± 0.4			
Platelet Count	PLA	242.2 ± 32.6	239.0 ± 37.3	0.85	0.91	0.55
	D100	243.4 ± 26.7	250.2 ± 26.5			
	D200	242.8 ± 23.9	242.0 ± 19.1			
	B500	247.4 ± 36.1	245.8 ± 30.9			
Abs Neutrophils (cells/uL)	PLA	2790 ± 1318	3046 ± 1295	0.45	0.88	0.38
	D100	2902 ± 719	2962 ± 949			
	D200	3926 ± 2324	3723 ± 2020			
	B500	2743 ± 737	2675 ± 702			
Abs Lymphocytes (cells/uL)	PLA	1893 ± 435	1805 ± 297	0.01	0.31	0.28
	D100	2068 ± 719	2173 ± 253			
	D200	1852 ± 528	1721 ± 149			
	B500	2268 ± 524	1899 ± 423			
Abs Monocytes (cells/μL)	PLA	477 ± 134	549 ± 168	0.92	0.04	0.007
	D100	494 ± 159	597 ± 225			
	D200	549 ± 152	504 ± 96			
	B500	545 ± 178	530 ± 205			
Abs Eosinophils (cells/μL)	PLA	218 ± 162	214 ± 162	0.17	0.09	0.62
	D100	179 ± 116	165 ± 96			
	D200	225 ± 148	188 ± 106			
	B500	183 ± 119	179 ± 161			
Abs Basophils (cells/μL)	PLA	42.8 ± 12.4	45.6 ± 10.9	0.47	0.94	0.67
	D100	38.4 ± 7.6	43.8 ± 12.2			
	D200	49.0 ± 18.5	44.6 ± 9.3			
	B500	41.2 ± 20.7	36.8 ± 11.6			
Neutrophils (%)	PLA	52.1 ± 13.2	52.2 ± 11.7	0.24	0.42	0.29
	D100	50.7 ± 7.3	49.1 ± 6.7			
	D200	56.7 ±14.4	57.3 ± 12.0			
	B500	47.5 ± 7.9	50.4 ± 7.8			
Lymphocytes (%)	PLA	34.7 ± 9.9	33.1 ± 7.8	0.17	0.26	0.32
	D100	36.7 ± 6.2	37.4 ± 5.2			
	D200	30.0 ± 9.5	29.8 ± 7.6			
	B500	39.5 ± 7.1	36.0 ± 4.7			
Monocytes (%)	PLA	8.5 ± 2.1	10.0 ± 3.1	0.12	0.15	0.60
	D100	9.0 ± 1.1	10.0 ± 2.9			
	D200	8.9 ± 2.7	8.8 ± 2.7			
	B500	9.3 ± 1.8	9.8 ± 2.4			
Eosinophils (%)	PLA	4.0 ± 3.0	3.9 ± 3.1	0.30	0.25	0.72
	D100	3.0 ± 1.4	2.7 ± 1.3			
	D200	3.6 ± 2.8	3.3 ± 2.5			
	B500	3.0 ± 1.5	3.1 ± 2.3			
Basophils (%)	PLA	0.8 ± 0.3	0.8 ± 0.3	0.37	0.43	0.95
	D100	0.7 ± 0.1	0.8 ± 0.3			
	D200	0.8 ± 0.3	0.8 ± 0.3			
	B500	0.7 ± 0.2	0.7 ± 0.2			

**Table 7:** Comprehensive metabolic panel.

Variable Name	Group	Pre	Post	Group (G) (*p*)	Time (T) *(p)*	G x T (*p*)
Glucose (mg/dL)	PLA	90.6 ± 5.3	65.6 ± 11.0	0.66	0.02	0.17
	D100	92.0 ± 5.7	68.8 ± 10.0			
	D200	81.2 ± 13.6	73.2 ± 20.1			
	B500	89.2 ± 4.2	73.8 ± 15.4			
Blood Urea Nitrogen (g/dL)	PLA	16.2 ± 2.3	15.6 ± 2.3	0.14	0.11	0.46
	D100	13.8 ± 1.5	13.8 ± 1.3			
	D200	15.4 ± 1.9	14.8 ± 2.2			
	B500	15.8 ± 2.2	15.2 ± 2.5			
Creatinine (mg/dL)	PLA	1.0 ± 0.1	1.0 ± 0.1	0.04	0.55	0.008
	D100	0.9 ± 0.1	1.0 ± 0.1			
	D200	1.0 ± 0.1	0.9 ± 0.1			
	B500	1.0 ± 0.1	0.9 ± 0.1			
Glomerular Filtrate Rate	PLA	107.2 ± 12.0	102.2 ± 12.8	0.03	0.30	0.02
	D100	115.6 ± 8.4	109.4 ± 8.8			
	D200	106.2 ± 13.0	111.0 ± 11.9			
	B500	106.6 ± 13.3	109.2 ±12.8			
Sodium (mM)	PLA	137 ± 1	139 ± 2	0.20	<0.001	0.82
	D100	136 ± 2	139 ± 2			
	D200	135 ± 2	137 ± 2			
	B500	136 ± 2	138 ± 3			
Potassium (mM)	PLA	4.3 ± 0.2	4.0 ± 0.1	0.36	0.007	0.90
	D100	4.2 ± 0.2	3.9 ± 0.2			
	D200	4.3 ± 0.2	4.0 ± 0.2			
	B500	4.2 ± 0.3	3.9 ± 0.2			
Chloride (mM)	PLA	102 ± 2	102 ± 1	0.45	0.02	0.18
	D100	103 ± 2	103 ± 2			
	D200	101 ± 2	103 ± 2			
	B500	101 ± 3	103 ± 2			
Carbon Dioxide (mM)	PLA	25 ± 2	26 ± 3	0.62	0.16	0.78
	D100	25 ± 1	26 ± 1			
	D200	26 ± 1	27 ± 2			
	B500	25 ± 1	26 ± 3			
Calcium (mg/dL)	PLA	9.3 ± 0.4	9.7 ± 0.2	0.26	0.12	0.049
	D100	9.4 ± 0.2	9.5 ± 0.3			
	D200	9.3 ± 0.5	9.4 ± 0.3			
	B500	9.5 ± 0.3	9.5 ± 0.3			
Total Protein (g/dL)	PLA	6.8 ± 0.4	6.9 ± 0.3	0.94	0.74	0.22
	D100	6.8 ± 0.3	6.9 ± 0.3			
	D200	6.8 ± 0.6	6.8 ± 0.4			
	B500	7.0 ± 0.5	6.8 ± 0.5			
Albumin (g/dL)	PLA	4.6 ± 0.2	4.5 ± 0.1	0.42	0.38	0.80
	D100	4.5 ± 0.1	4.4 ± 0.2			
	D200	4.5 ± 0.2	4.4 ± 0.0			
	B500	4.6 ± 0.1	4.5 ± 0.1			
Globulin (g/dL)	PLA	2.2 ± 0.3	2.3 ± 0.3	0.62	0.26	0.17
	D100	2.3 ± 0.3	2.4 ± 0.3			
	D200	2.4 ± 0.4	2.4 ± 0.3			
	B500	2.4 ± 0.4	2.3 ± 0.5			
Alb/Glob Ratio	PLA	2.1 ± 0.2	2.0 ± 0.2	0.37	0.11	0.28
	D100	2.0 ± 0.3	1.8 ± 0.3			
	D200	1.9 ± 0.3	1.9 ± 0.3			
	B500	1.9 ± 0.3	2.0 ± 0.4			
Total Bilirubin (mg/dL)	PLA	0.9 ± 0.3	0.8 ± 0.3	0.52	0.14	0.37
	D100	0.8 ± 0.3	0.8 ± 0.3			
	D200	0.9 ± 0.4	0.8 ± 0.3			
	B500	1.0 ± 0.4	0.9 ± 0.4			
Alkaline Phosphatase (U/L)	PLA	53 ± 10	51 ± 10	0.13	0.16	0.45
	D100	51 ± 11	52 ± 10			
	D200	51 ± 13	50 ± 10			
	B500	54 ± 12	53 ± 13			
AST (U/L)	PLA	22 ± 5	22 ± 5	0.22	0.18	0.55
	D100	24 ± 7	24 ± 7			
	D200	24 ± 6	23 ± 5			
	B500	20 ± 3	20 ± 3			
ALT (U/L)	PLA	19 ± 6	19 ± 5	0.93	1.00	0.60
	D100	19 ± 7	20 ± 7			
	D200	19 ± 4	18 ± 3			
	B500	18 ± 6	18 ± 5			

## Data Availability

Data and/or statistical analyses are available upon request on a case-by-case basis for non-commercial scientific inquiry and/or educational use.
